# A review of antimicrobial stability testing guidance for outpatient parenteral antimicrobial therapy programmes: is it time for global harmonization of testing frameworks?

**DOI:** 10.1093/jacamr/dlae186

**Published:** 2024-11-28

**Authors:** Saiyuri Naicker, Jason A Roberts, Vesa Cheng, Suzanne L Parker, R Andrew Seaton, Mark Gilchrist, Fekade B Sime

**Affiliations:** The University of Queensland Centre for Clinical Research, University of Queensland, Brisbane, Queensland, Australia; The University of Queensland Centre for Clinical Research, University of Queensland, Brisbane, Queensland, Australia; Herston Infectious Diseases Institute (HeIDI), Metro North Health, Brisbane, Australia; Departments of Pharmacy and Intensive Care Medicine, Royal Brisbane and Women’s Hospital, Brisbane, Australia; Division of Anaesthesiology Critical Care Emergency and Pain Medicine, Nîmes University Hospital, University of Montpellier, Nîmes, France; The University of Queensland Centre for Clinical Research, University of Queensland, Brisbane, Queensland, Australia; Department of Anaesthesia and Intensive Care, Faculty of Medicine, Chinese University of Hong Kong, Hong Kong, China; Medical Education Unit, Princess Alexandra Hospital, Metro South Health, Brisbane, Australia; The University of Queensland Centre for Clinical Research, University of Queensland, Brisbane, Queensland, Australia; Department of Infectious Diseases, Queen Elizabeth University Hospital, Glasgow, UK; Department of Pharmacy/Infection, Imperial College Healthcare NHS Trust, London, UK; Department of Infectious Diseases, Imperial College London, London, UK; The University of Queensland Centre for Clinical Research, University of Queensland, Brisbane, Queensland, Australia

## Abstract

Antimicrobial stability is an important consideration for treatment planning and service delivery in outpatient parenteral antimicrobial therapy (OPAT) programmes. Regulation of stability assessment varies by region, and conflicting guidance and standards exist. This leads to disparity of equity in access and limits availability of certain antimicrobials for managing infections in the outpatient setting. This review discusses the degree to which the international regulatory bodies have reached consensus on the regulation of antimicrobial stability testing, specifically for OPAT, and describes the variation in antimicrobial recommendations across regulatory bodies. The three major findings in this review are (i) variation in antimicrobial stability testing guidance, particularly in relation to temperature; (ii) lack of regulatory guidance, specifically in that some regions did not have OPAT guidelines; and (iii) only the UK’s NHS has provided non-regulatory OPAT-specific advice on antimicrobial stability testing. In conclusion, harmonization of antimicrobial stability testing to form a global OPAT-specific regulatory framework, particularly considering ‘areas of variation’ amongst current guidance, is required. We call for the development of a global OPAT antimicrobial stability testing framework with consensus from accepted antimicrobial stability criteria, expert opinion and pharmacopoeial best practice.

## Introduction

Outpatient parenteral antimicrobial therapy (OPAT) is an accepted modality of care for medically stable patients who require IV antimicrobial treatment without hospital admission or to support early discharge.^1^ In addition to providing patients with care closer to home, OPAT is highly cost-effective^2,3^ and reduces hospital-associated infection risk.^4,5^ OPAT has been used safely and effectively to treat a very wide range of infections including osteomyelitis, cellulitis, bronchiectasis, intra-abdominal infections, drug-resistant urinary tract infections and infective endocarditis.^6^ Lack of antimicrobial stability data in a patient-worn device has significant implications for the choice and stewardship of antimicrobials available in OPAT and may be a barrier to provision of non-inpatient treatment. The WHO has defined drug stability as ‘The ability of a pharmaceutical product to retain its chemical, physical, microbiological and biopharmaceutical properties within specified limits throughout its shelf-life.’ Some antimicrobials are unstable at higher temperatures and may undergo rapid degradation leading to formation of biologically inactive or potentially toxic compounds, which can also potentially affect the infusion flow in the devices.^7^ In hospitals (in advanced healthcare systems) infusion solutions and pumps are separated from the body and therefore are not subject to the increased temperature exposure that occurs in the outpatient settings, whereas ambulatory devices are usually worn in close proximity to the body, subjecting them to body temperature.

Due to the lack of published, open-access stability data for OPAT, the BSAC established an OPAT drug stability testing programme as part of its wider UK OPAT Initiative. This programme required that data be published in accordance with the UK’s NHS ‘yellow-covered document’ (YCD): ‘A standard protocol for deriving and assessment of stability: Part 1—aseptic preparations (small molecules)’.^8–10^ Several systematic reviews have collated OPAT UK NHS YCD-compliant studies to identify suitable antimicrobials for OPAT. However, to our knowledge, there has been no review on the consistencies between international stability testing guidelines.^11,12^ Improving consistency between guidelines that are grounded on maintaining scientific integrity and validity may increase the number of therapeutic options available for OPAT.^13,14^ This review will describe differences and similarities in existing regulations and identify areas that may benefit from global harmonization.

## Principles of antibiotic therapy

Antibiotics treat bacterial infections by either killing or inhibiting bacterial growth. As such, the effect of the drug on the bacteria, also known as pharmacodynamics, is important for optimizing treatment outcomes.^15^ Appropriate antibiotic selection requires consideration of many important factors, which may include: mode of action, expected susceptibility of the bacteria to the antibiotic and patient-related factors.^16^

Antibiotic resistance may occur when bacteria are exposed to subtherapeutic antibiotic concentrations. Therefore, antibiotics that kill or inhibit the most bacteria in the shortest period of time are most desirable.^15^ Once the appropriate antibiotic has been selected, consideration for the dose required to achieve the ideal concentration (exposure) at the site of infection is required. This is achieved through understanding the interplay between the administered drug and the patient’s body, i.e. pharmacokinetics.^15,16^ Antibiotic dosing is usually informed using therapeutic guidelines, the evidence for which is supported by clinical pharmacokinetic studies and clinical outcome trials.^17^ Appropriate antibiotic exposure is vital to good patient outcomes including mortality, success of treatment and suppression of emergence of resistance.^18^

Key factors influencing antimicrobial agent selection in OPAT are dosing frequency, drug stability and likelihood of toxicity.^19^ Even small modulations in antimicrobial exposure have been shown to result in significant effects on patient outcomes.^18^ Stability of drugs during storage and infusion is important as degradation reduces therapeutic drug exposure and potentially increase exposure to toxic degradation products.^20^

## Regulatory requirements for stability studies

Antimicrobials used in OPAT must maintain stability for the duration of storage following compounding, transportation and during administration,^7^ with data being available to support and assure their use in this setting.^21^

Regulatory requirements set by authorities must be followed for antimicrobials to gain market authorization and approval for use in prescribed indications. Despite storage and administration methods for antimicrobial infusions in OPAT programmes being so different to those within hospitals, many regulatory bodies only provide stability testing guidance relevant to hospital settings, without accounting for the fundamental differences of outpatient settings. Stability testing guidance materials include the US Pharmacopeial (USP) monographs regulated by the US FDA. In Europe guidance is provided by the EMA,^22^ in Australia by the Australian Therapeutic Goods Administration (TGA),^23^ some regions globally follow the WHO^24^ guidance and most of South-East Asia follows the guidance provided by the Association of South-East Asian Nations (ASEAN).^25^ These guidance documents are directly derived from or informed by the International Council for Harmonisation (ICH) of Technical Requirements for Pharmaceuticals for Human Use.^26^ The only authority that provides specific guidance on stability considerations in OPAT settings is the UK NHS document titled ‘Guidance on pharmaceutical issues concerning OPAT’. To the best of our knowledge, this is the only document that provides guidance on stability testing and acceptance criteria specifically in the OPAT setting. The document serves as a supplementary guidance in addition to the UK’s NHS YCD: ‘A standard protocol for deriving and assessment of stability: Part 1—aseptic preparations (small molecules)’.

Comparison of the available existing antimicrobial stability regulatory requirements allows an assessment of the differences between guidance, which may be used to inform and encourage an OPAT-specific global consensus on stability testing and evaluation.

## General stability testing regulation

Many factors affect stability of antimicrobials and, as such, regulatory bodies have defined the minimum requirements needed for drugs to be approved for OPAT use. The ICH has published the global standard for testing for drug stability. The ICH original guidance documents ‘Stability testing of new drug substances and products Q1A (R2)’,^26^ ‘Stability testing: photostability of new drug substances and products Q1B’^27^ and ‘Specifications: test procedures and acceptance criteria for new drug substances and new drug products: chemical substances Q6A’^28^ have provided a backbone to regulate drug stability testing. Regulatory bodies such as the FDA, TGA, ASEAN and EMA have published guidelines based on the ICH recommendations and incorporated geographically specific information (see Tables 1 and 2). However, the guidelines all refer to the original ICH recommendations and are based on the guidance in the ICH Q1A–Q1F stability guidance document series.^26^ The ICH has not released OPAT-specific recommendations and, as such, clinicians can only refer to these guidelines for in-hospital infusions. The ICH has proposed revisions of the guidance on stability in their concept paper published in 2022, and aims to have an update made available in 2025.^29^ This revision may include additional guidance on drug stability within infusion containers that is relevant to outpatient settings as well as OPAT temperature considerations for body-worn devices.

**Table 1.** Differences in regulatory requirements between world drug stability testing regulatory bodies considering testing frequency and storage conditions

Regulatory body	Testing frequency			General storage			Refrigerated		Freezer
	Long-term	Intermediate^a^	Accelerated^a^	Long-term	Intermediate	Accelerated	Long-term	Accelerated (room temp.)	Long-term
ICH, FDA (US), TGA, EMA	3 mo in first year, 6 mo second year, then annually	4 timepoints	3 timepoints	25 ± 2°C 60 ± 5% RH OR 30 ± 2°C 65 ± 5% RH	30 ± 2°C 65 ± 5% RH	40 ± 2°C 75 ± 5% RH	5 ± 3°C	25 ± 2°C 60 ± 5% RH	−20 ± 5°C
WHO	3 mo in first year, 6 mo second year, then annually	4 timepoints	3 timepoints	25/30 ± 2°C 60/65/75 ± 5% RH	30 ± 2°C 65 ± 5% RH	40 ± 2°C 75 ± 5% RH	5 ± 3°C	25/30 ± 2°C 60/65/75 ± 5% RH	−20 ± 5°C
UK NHS (YCD)	4 timepoints (minimum 3 replicates)			25/32/37 ± 2°C	32/37 ± 2°C	40 ± 2°C	5 ± 3°C		−20 ± 5°C
ASEAN	3 mo in first year, 6 mo second year, then annually	3 timepoints		30 ± 2°C 75 ± 5% RH 30 ± 2°C RH not specified	40 ± 2°C 75 ± 5% RH or at more stressful conditions for stress testing		5 ± 3°C	25 ± 2°C 60 ± 5% RH	−20 ± 5°C

ASEAN, Association of South-East Asian Nations; ICH, International Council for Harmonisation of Technical Requirements for Pharmaceuticals for Human Use; RH, relative humidity; TGA, Therapeutic Goods Administration of Australia; YCD, ‘yellow-covered document’.

^a^Timepoints minimum.

**Table 2.** Differences in regulatory requirements considering container permeability, labelling and acceptance criteria

Regulatory body	Documents referenced	Impermeable containers	Semi-impermeable containers			Labelling	Acceptance criteria
			Long-term	Intermediate	Accelerated		
ICH, TGA	Stability testing of new drug substances and products Q1 A (R2); Stability testing: photostability of new drug substances and products Q1B; Specifications: test procedures and acceptance criteria for new drug substances and new drug products: chemical substances Q6A	Any controlled or ambient humidity condition	25 ± 2°C, 40 ± 5% RH or 30 ± 2°C, 35 ± 5% RH	30 ± 2°C 65 ± 5% RH	40 ± 2°C ≤ 25% RH	Label should be specific to the demonstrated stability avoiding the use of vague terms in accordance with national/regional requirements	5% labelled content of the drug
EMA	Note for guidance on stability testing: stability testing of new drug substance and products	Any controlled or ambient humidity condition	25/35 ± 2°C 40/35 ± 5% RH	30 ± 2°C 65 ± 5% RH	40 ± 2°C ≤ 25% RH	Label should be specific to the demonstrated stability avoiding the use of vague terms in accordance with national/regional requirements	Refer to drug-specific monograph if it exists or 5% labelled content of the drug
WHO	Annex 10: Stability testing of active pharmaceutical ingredients and finished pharmaceutical products	Any controlled or ambient humidity condition	25 ± 2°C, 40 ± 5% RH or 30 ± 2°C, 35 ± 5% RH (can be altered based on climatic conditions)	30 ± 2°C 35 ± 5% RH	40 ± 2°C ≤ 25% RH	Label should be specific to the demonstrated stability avoiding the use of vague terms in accordance with national/regional requirements	5% labelled content of the drug
UK NHS (YCD)	A standard protocol for deriving and assessment of stability: Part 1—aseptic preparations (small molecules)	Permeability of the container should be assessed for critical physical properties including oxygen permeability, water loss, light permeability, material constitution and adsorbent potential. Specific guidelines are provided for syringes, infusion containers, ambulatory infuser devices, glass vials and eye dropper bottles				Not stated	95%–105% of stated amount
ASEAN	ASEAN guideline on stability study of drug product (R1)	Any controlled or ambient humidity condition	30 ± 2°C 35 ± 5% RH	Not stated	40 ± 2°C ≤ 25% RH	Label should be specific to the demonstrated stability avoiding the use of vague terms in accordance with national/regional requirements	5% change in assay from its original value

ASEAN, Association of South-East Asian Nations; ICH, International Council for Harmonisation of Technical Requirements for Pharmaceuticals for Human Use; RH, relative humidity; TGA, Therapeutic Goods Administration of Australia; YCD, ‘yellow-covered document’.

Although similar to the ICH guideline, the WHO’s guidance document ‘Annex 10—Stability testing of active pharmaceutical ingredients and finished pharmaceutical products’,^24^ provides more details and consolidates the three ICH guidelines.^26–28^ All the guidelines are similar in requiring: (i) the testing of three primary batches, (ii) testing of the product container closure systems during storage and administration, and (iii) stress testing under similar conditions, except for the ASEAN guidelines.^25^ The UK’s NHS YCD ‘A standard protocol for deriving and assessment of stability: Part 1—aseptic preparations (small molecules)’ differs from the other guidelines in that (i) it provides highly specific testing protocols, (ii) it mandates some testing (see Table 3), (iii) the testing frequency guidelines are less complex and restrictive, and therefore more achievable (Table 1), and (iv) it has a requirement for reproducibility. On the other hand, the UK’s NHS YCD permeability storage condition testing requirements are non-specific stating: ‘assessed as deemed appropriate according to the device being used’^10^ and there are no stated labelling requirements (Table 2).

**Table 3.** Differences in regulatory guidelines for drug stability testing

Regulatory body	Stress testing	Container closure system	Concentration/dose	Specific tests	References
ICH, FDA (US), ASEAN, TGA, EMA	Temperature, humidity, oxidation, photolysis, pH stability, photostability	Packaging proposed for dosage (including multidose), include data on all the preparations of the drug including various delivery devices with justifications if extrapolated	Simulated use of drug in practice	Physical, chemical, biological, microbiological, preservative, functionality	^22,23,25–28,30^
WHO	Temperature, humidity, oxidation, photolysis, pH stability, photostability, cyclic studies, freeze-thaw	Packaging proposed for dosage (including multidose), include data on all the preparations of the drug including various delivery devices with justifications if extrapolated	Simulated use of drug in practice (contains section on in-use and hold-time stability)	Physical, chemical, biological, microbiological, preservative, functionality	^24,31^
UK NHS (YCD)	Temperature, humidity, oxidation, photolysis, pH stability, photostability	Packaging proposed for dosage unless extrapolation to alternative dosage forms is relevant and acceptable	Multiple dosing regimens as well as simulated in use	Colour, clarity and precipitation, pH, active pharmaceutical ingredient (API) concentration, subvisible particle counts, degradation product concentration, moisture loss, container extractables and leachable, excipient concentrations	^10^

ASEAN, Association of South-East Asian Nations; ICH, International Council for Harmonisation of Technical Requirements for Pharmaceuticals for Human Use; RH, relative humidity; TGA, Therapeutic Goods Administration of Australia; YCD, ‘yellow-covered document’.

## Stability testing requirements

Table 3 shows limited differences between the technical testing requirements for stability, as far as the assessments for stability are concerned. Overall, the stress testing requirements from the WHO include assessing temperature, humidity, oxidation, photolysis, pH-stability and photostability with the addition of cyclic studies and freeze-thaw stability.^24^ For specific tests on stability, the UK’s NHS YCD ‘A standard protocol for deriving and assessment of stability: Part 1—aseptic preparations (small molecules)’ is more prescriptive than the other guidance documents, requiring additional specific testing of colour, clarity and precipitation, pH, active pharmaceutical ingredient (API) concentration, subvisible particle counts, degradation product concentration, moisture loss, container extractables and leachable excipient concentrations.^10^ The ICH ‘Stability testing of new drug substances and products Q1A (R2)’, the WHO’s ‘Annex 10—Stability testing of active pharmaceutical ingredients and finished pharmaceutical products’ and their related guidances lack specificity, with recommendations to take ‘appropriate testing’.^24,26^ A key difference in testing requirements relates to dosage. The UK’s NHS YCD requires a variety of different dose concentrations be tested;^10,24^ however, the ICH and its related guidance documents recommend simulating the use of the drug in practice, not specifically requiring the testing of varying doses.^24,26^

## Testing frequency and storage testing requirements

Until the early 2000s, the WHO guidance did not specifically account for the variation in storage temperature and humidity in different geographical regions. The WHO has since released an update to the stability conditions for long-term storage testing that accounts for variance that may occur globally, listing variances by country.^31^ This is another key area of stability testing guidance that has yet to be globally harmonized. The aforementioned document ‘Annex 10—Stability testing of active pharmaceutical ingredients and finished pharmaceutical products’ is the WHOs first step towards global harmonization. However, it has yet to be widely adopted. The UK’s NHS^10^ and several other non-member states of the ICH^32^ have their own guidance. A survey reported by the Centre for Innovation in Regulatory Science (CIRS) found that observers (non-member states) of the ICH were implementing the drug stability testing regulatory guidelines with modifications.^32^ Future harmonization of the regulatory framework regarding storage testing requirements should be informed by the modifications made by these observers and varying conditions published in the WHO guidance^31^ (Table 1).

## API concentration

Table 2 compares the stability acceptance criteria cited by all the relevant authorities for the regulation of stability testing. The EMA guidance has stated that all active substances must comply with European Pharmacopoeia (EP)^33^ or pharmacopoeia of a European Union Member State monographs, noting that the British Pharmacopoeia (BP) is part of the European Union member state monographs. However, where these monographs do not exist, stability testing must occur in order to determine the acceptance criteria.^22^ The EMA guidance does not specify limits for API degradation for stability testing, stating that specifications according to the monographs must be met.^22^

The ICH has defined the API acceptance criterion for stability as a lack of ‘significant change’, which is defined as a 5% change from the original value.^34^ This has been adopted across multiple regions and guidances including the ASEAN,^25^ TGA^23^ and FDA.^30^ All of these regulatory authorities refer directly to the ICH Q1A (R2) harmonized tripartite guideline on ‘Stability testing of new drug substance and products’.^26^

The WHO guidance uses a similar approach to the ICH. Using slightly more definitive language stating: ‘In general, “significant change” for a finished pharmaceutical product (FPP) is defined as: a change from the initial content of API(s) of 5% or more detected by assay.’^24^ The UK's NHS YCD ‘A standard protocol for deriving and assessment of stability: Part 1—aseptic preparations (small molecules)’ requires that acceptance criteria be set to no more than 5% loss of API unless loss of up to 10% of API can be justified by reference to specified limits set by the BP.^10,35^ These different thresholds can result in contrasting recommendations, which can potentially affect antimicrobial management and patient equity of access options in the clinical setting. However, the clinical significance of 5% versus 10% API loss remains unclear. For most antimicrobials, a 5%–10% API loss may not be significant given this threshold comfortably exists within routinely observed between-patient pharmacokinetic variation.^36^ There is significant evidence of the benefits of continuous infusions in critically ill patients; however, the outcomes of use in OPAT are unknown.^37^ Nevertheless, for those antimicrobials where degradation products are toxic, the effect may be clinically relevant and the patient effects worth investigation.

## Harmonization of stability testing guidelines

The responsibility for providing evidence of the stability of antimicrobials for use in OPAT is distributed among several key stakeholders: pharmaceutical companies, academia and primary care providers such as hospitals.^38^ Pharmaceutical companies should conduct stability studies and provide comprehensive dosing information, including data on the stability of antimicrobials, which is governed under a harmonized regulation.^39^ Academia, comprising clinical researchers and academic institutions, has a critical role in conducting independent clinical trials and stability studies within OPAT settings to generate real-world evidence regarding the efficacy, safety and stability of antimicrobials.^40^ Finally, whereas healthcare providers such as hospitals are responsible for the implementation of OPAT programmes, monitoring patient outcomes and potentially collaborating in research, they generally lack the specialized laboratories and expertise required to conduct formal stability experiments.^38^ Such experiments typically require controlled environments and standardized protocols. In summary, although hospitals can contribute to real-world evidence through patient monitoring and adherence to guidelines, formal stability testing is generally conducted by pharmaceutical companies or academic institutions under the guidance of regulatory agencies.

In 2005, the ASEAN guidelines were harmonized with the global stability testing requirements. However, these have been revised twice,^25^ resulting in more harmonization with the ICH regulations.^41^ In March 2017, the Australian TGA replaced all stability guidelines to reference either the EMA or the ICH documents,^23^ harmonizing its stability testing regulatory framework with the ICH. The WHO’s stability guidelines were prepared in consultation with the ICH guidance but continue to differ in long-term storage guidance. Due to this, the ICH has withdrawn guidance on storage in geographical regions with a hotter and more humid climate, deferring back to the WHO.^24,31^ Although the ICH stability testing guidance documents remained relatively unchanged since 2003, the ICH published a concept paper in response to the CIRS report.^32^ The concept paper details a planned revision of the guidance documents that may address many of the variations raised in this review as well as the CIRS report to bring about a consensus. Additionally, the planned revision of the ICH stability testing guidance is expected to include specific guidance for outpatient settings.^29^ It is not yet known to what extent the guidance will encompass all regulatory requirements for OPAT drugs or its sufficiency to support clinicians and manufacturers in providing antimicrobials that are safe and effective in OPAT.^8,10^

## Antimicrobial stability for OPAT regulatory requirements

All major regulatory authorities apart from the UK’s NHS YCD provide no specific guidance on stability testing for the outpatient setting. The TGA’s stability testing for prescription medicines guidance^23^ comments on the testing of delivery or administration devices, specifically in relation to the regulation of inhaled and nasal preparations, not parenteral drugs. Similarly, the ASEAN guidance has specifically referred to device testing for drug admixture and the potential for drug–device interactions, without specifying the type of devices or testing parameters or the nature of stability testing required.^25^ Although certain aspects of stability testing render it reasonable to assume similarities between general and OPAT-related stability (such as doses, batch testing and even certain acceptance criteria), the OPAT stability testing protocols should be more specific to include conditions that are expected to occur in outpatient settings. These conditions may include the specific body-worn ambulatory devices used in OPAT, preference for alternate regimens of drug delivery (intermittent or continuous infusion), and variation in storage and ‘in-use’ temperatures conditions that may lead to unpredictable drug degradation.

The UK’s NHS is the regulatory authority that includes a specific section describing guidance for OPAT: ‘Guidance on the pharmaceutical issues concerning OPAT services and other outpatient intravenous therapies’.^8^ This document supplements the UK’s NHS YCD, ‘A standard protocol for deriving and assessment of stability: Part 1—aseptic preparations (small molecules)’^10^ as well as the criteria provided by the British Pharmacopoeia with additional OPAT-specific advice.^35^ The main requirements for stability testing of antimicrobials used in OPAT are (i) suitability of various storage conditions, (ii) stability while in use, e.g. drug-device considerations, (iii) study of degradation products, (iv) physical tests and (v) extrapolation of data.

## Temperature stability for OPAT

The UK’s NHS ‘Guidance on the pharmaceutical issues concerning OPAT services and other outpatient intravenous therapies’^8^ stability requirements states antimicrobials must be subjected to storage at refrigeration temperatures (5 ± 3°C) where possible or under recommended conditions when solubility is a concern^10^ for a maximum storage period prior to exposure to the ‘in-use’ temperature at 32°C.^8^ Maximum storage period in both refrigeration or storage conditions and the sequential elevated temperature for ‘in-use’ stability must be assessed. For OPAT, testing is required to closely mimic the temperature conditions that drugs will be exposed to during administration via infuser next to the patient (e.g. at 32°C).^8,20^ If the infusion period exceeds 1 h, the in-use stability must be assessed for the length of time the drug is expected to be exposed to the elevated temperature during administration.^8^

## Physical stability requirements for OPAT

Physical examinations are required for OPAT stability testing to assess and report physical changes that may indicate loss of stability.^8^ OPAT guidance from the UK’s NHS ‘Guidance on the pharmaceutical issues concerning OPAT services and other outpatient intravenous therapies’ stability requirements states a minimum requirement of examinations to include the testing for the presence of subvisible particles and the assessment of pH stability.^8^ Any additional physical examinations may follow general stability requirements described in the UK’s NHS YCD ‘A standard protocol for deriving and assessment of stability: Part 1—aseptic preparations (small molecules)’.^10^ The UK’s NHS also requires that, where there is guidance provided in a monograph, the British Pharmacopoeia monographs (non-EU) be followed thoroughly.^35^

When comparing the general stability requirements across the regulatory authorities, the UKs NHS YCD ‘A standard protocol for deriving and assessment of stability: Part 1—aseptic preparations (small molecules)’^10^ provides the most comprehensive description of the specific physical tests recommended, summarized above and in Tables 1,4.^8,10^ However, the ICH outlines specific tests to be performed for parenteral drug products. This includes physical tests, i.e. pH and particle sizing, which are more related to batch testing for good manufacturing practice, rather than stability testing for the purposes of outpatient use.^34^ The WHO in their general guidance on stability have noted that colour, clarity, particulate matter, pH, sterility and endotoxins should be assessed, but do not give specific testing guidance for outpatient settings.^24^ The requirements for the physical examinations for stability testing globally are generally consistent (Table 3) and as such, harmonization may be simple to achieve by providing a consensus of the physical tests required, associated protocols and acceptance criteria.

**Table 4.** Differences in regulatory requirements for drug stability in general versus OPAT drug stability as per UK NHS YCD

Regulation	General stability requirements	OPAT-specific stability requirements as per UK NHS YCD
Refrigerator stability	5 ± 3°C (room temp. 25 ± 2°C)	5 ± 3°C
‘In-use’ temperature stability	Stated as ‘near’ body temperature 32 ± 1°C	32°C
Time period	Maximal storage time, triplicate	‘In-use’ stability must be assessed for duration of therapy depending on the infusion type
Study of degradation products	Must be assessed and investigated using a validated bioanalysis method	Analysis and understanding of degradation products
Physical examination	Colour, clarity and precipitation checked against standards set by BP	Stability-indicating assay and analysis must be done to ascertain stability
Subvisible particle counts	Light obscuration technique including particle size	Light obscuration technique including particle size
pH stability	Changes in pH are to be investigated as indicative of other changes that need further investigation	Assess and report any significant changes in pH
Data	Reaction kinetics, its solubility, and its ability to adsorb to surfaces are all important considerations that require an expert opinion before a decision is made to extrapolate	Expert interpretation of data for extrapolation when required
Acceptance criteria	Loss of 5% of API constitutes maximum shelf life, BP specifications are to be followed. 10% loss may be acceptable with justification and assessment of degradation products^10^	Where there is a BP monograph the product must be fully compliant with the BP

API, active pharmaceutical ingredient; BP, British Pharmacopoeia; YCD, ‘yellow-covered document’.

## Regulatory guidance on elastomeric device selection

Current regulatory guidance, such as the UK NHS YCD,^10^ ICH Q1A^26^ and FDA^30^ recommendations summarized in Table 2, primarily focuses on the stability of drugs in their packaged forms, under controlled environmental conditions like temperature, humidity and light exposure. These regulations ensure that drugs retain their efficacy, safety and potency throughout their shelf life. However, when it comes to antimicrobials delivered via elastomeric pumps, the existing guidance does not specifically address the unique challenges posed by using these devices in real-world clinical settings, particularly for prolonged infusion therapy in outpatient environments.

Elastomeric devices are commonly used for outpatient antimicrobial therapy, offering benefits like continuous drug delivery over extended periods.^42^ However, the materials used in these devices, such as silicone or rubber, can interact with the drugs, leading to adsorption and degradation.^42^ Regulatory bodies do not explicitly mandate the evaluation of drug stability within these devices, leaving the request for this information to the responsibility of the healthcare providers. As a result, clinicians often rely on limited data or device manufacturer recommendations, which may not fully capture the stability risks of different antimicrobials when used with different elastomeric devices.

Additionally, it is essential to expand stability testing to include investigations to measure the potential interaction between antimicrobials and elastomeric devices. Regulatory agencies should require device-specific stability studies that replicate real-use conditions, such as prolonged infusion durations typical in OPAT, and environmental fluctuations that occur during patient use. These studies should assess adsorption to elastomeric surfaces, temperature effects, and the impact of infusion time on the drug’s potency and efficacy.^43,44^ By incorporating these factors into regulatory requirements, the selection of elastomeric devices could be better informed, ensuring that the chosen devices minimize drug sequestration and maintain therapeutic concentrations throughout the infusion period.

Additionally, clearer guidelines on labelling and device compatibility should be developed. Regulatory submissions should include detailed data on the stability of antimicrobials when administered through elastomeric devices, allowing clinicians to make informed decisions regarding device selection. This would improve the safety and efficacy of OPAT, reducing the risks of subtherapeutic dosing and resistance development.^43^ Subtherapeutic dosing is known to be associated with a reduction in pharmacokinetic target attainment, which is vital to improving patient outcomes with antimicrobial therapy.^45^ By expanding regulatory frameworks to address device-specific antimicrobial stability, the quality of antimicrobial delivery in OPAT settings can be enhanced, potentially leading to improved clinical results. This aligns with national action plans, such as the UK’s 20 year vision for antimicrobial resistance^46^ and the US National Action Plan for Combating Antibiotic-Resistant Bacteria,^47^ which emphasize optimizing antimicrobial use to reduce antimicrobial resistance and improve patient care.

## Data management for OPAT

The UK’s NHS ‘Guidance on the pharmaceutical issues concerning OPAT services and other outpatient intravenous therapies’ document specifies that expert interpretation and full knowledge and understanding of the product and components are required for data extrapolation to be acceptable. Whereas the OPAT-specific requirements regarding data extrapolation are clarified by the UK’s NHS YCD ‘A standard protocol for deriving and assessment of stability: Part 1—aseptic preparations (small molecules)’, other data management requirements for OPAT stability testing are not differentiated from general stability testing requirements.^8^ The WHO guidelines and the ICH guidance on general (not OPAT) stability provide a detailed recommendation of statistical methodology that should be undertaken. However, this rigid methodology may be difficult to apply to OPAT preparations due to the variability in container materials used.^24,27,30^ Comparatively, the guidance on data extrapolation provided by the UK’s NHS YCD is more generic than other guidance documents and can therefore be more easily adapted to a range of conditions that may be encountered, including those of outpatient settings.^8^

## Assessment of degradation products for OPAT

Currently all stability testing regulations require stress testing, which may result in the qualitative assessment of the degradation product. The regulations do not specifically require that the degradation products be identified or quantified, only that stress testing be performed to characterize degradation. In the context of OPAT drug degradation, the primary focus for stability testing is in relation to the increased risk of exposure to elevated temperatures that could result in degradation of the API.^22–25,30^

Both general stability and OPAT-specific requirements state that the presence of degradation products must be assessed in all samples using a validated bioanalysis assay. Descriptions of degradation products need to be provided, as presence of toxic degradants limits the expiry period.^8^

The major differences between the documents lies in the acceptance criteria that the degradation products are measured against. Within the ICH-aligned stability guidelines, apart from the FDA guidance on stability, the European pharmacopoeial monographs^33^ are the primary referenced pharmacopoeial monographs, whereas for the UK’s NHS YCD it is the British monographs,^35^ and for the US FDA the USP monographs.^48^ This poses a regulatory anomaly and challenge for clinical practice in that the European, British and US pharmacopoeias each may provide a different criterion to evaluate stability. In the case of ceftazidime, the toxic degradation product is pyridine. The previous USP editions had the limit of 1.1 mg/mL allowable in solutions, which provided scope to include clinically relevant infusions; however, more recent editions^48^ have a 0.4% or 0.3% (w/w) limit of pyridine in the ceftazidime formulations containing sodium carbonate or arginine, respectively, neither of which are clinically commonly used infusions,^44^ whereas the BP and EP have the limits based on peak area obtained from a specific validated bioanalytical assay conducted using specific pyridine controls.^33,35^ This has been the cause of debate among those seeking to publish stability data pertaining to ceftazidime stability^49^ or evaluate it.^11^ The variation between the pharmacopoeias warranted a study by Kameyama *et al.*^50^ in which the harmonization of three major pharmacopoeias was assessed. The study found several major areas of variation: the monographs themselves, the assay and impurities sections, and physical tests relating to stability. Although the study did not focus on the BP, it compared the EP with the Japanese and US ones, providing valuable insight to the current state of disharmony between the pharmacopoeias.^50^ It is therefore clear that harmonization of the pharmacopoeias would need to occur for there to be useful global guidance on stability testing procedures and acceptance criteria for degradation products.

## Evaluating current OPAT stability evidence

**Figure 1.**
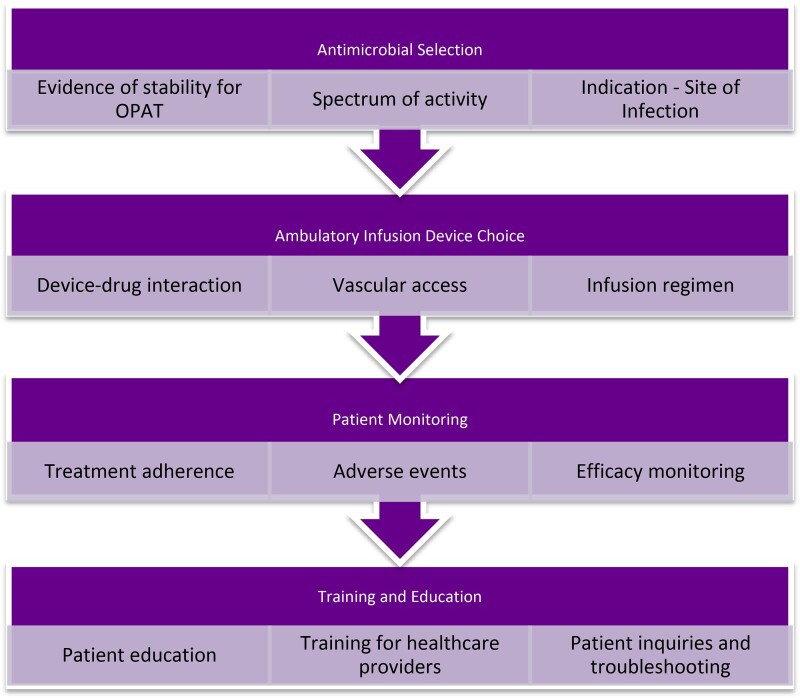
Schematic representation of key considerations for antimicrobial use in OPAT programmes.

The need for open-access OPAT antimicrobial stability data to support best OPAT practice prompted the BSAC to launch the drug stability programme (DSP) in 2019 as part of its wider UK OPAT Initiative. This was further prioritized in the OPAT Strategy 2022–2025.^51^ The strategy included supporting the need for open-access drug stability data, the provision of educational materials for the practice of evidence-based OPAT, as well as the publication of best practice guidelines.^9,51^ Over the past decade, systematic reviews on the stability of antimicrobials for OPAT have highlighted the critical need for careful antimicrobial selection based on stability under varying storage conditions.^7,11–13^ Key factors affecting the stability of antimicrobials like β-lactams, glycopeptides and aminoglycosides include temperature, diluent type and storage duration. Despite consistent calls for standardized protocols in OPAT, systematic reviews have applied different stability criteria, largely due to the absence of an international consensus on OPAT stability testing. The UK’s NHS YCD OPAT guidelines are regarded as the most explicit,^7^ yet many reviews have not adopted them as the standard for OPAT stability data.^11^ This is often attributed to differences in geographical climates and the adoption of local governance, which takes precedence over a global standard. In some cases, authors found the UK’s NHS YCD guidelines too restrictive and opted for less stringent criteria to allow more therapeutic options for OPAT settings.^12,13^ This variability has created uncertainty and poses challenges to antimicrobial stewardship, especially given the growing concerns over antimicrobial resistance.^4^ Based on the variables discussed in this review Figure 1 summarizes a suggested schematic framework for the implementation of current OPAT regulatory guidance to aid design of stability studies as well as clinical decision-making regarding OPAT, particularly highlighting key decision-making points at which considerations of stability could be regulated.

## Concluding remarks

This review of the global regulatory framework regarding stability testing has found that no comprehensive guidelines exist for drugs used in OPAT settings. General recommendations from the UK’s NHS YCD^8^ may be used and adopted in future iterations of the ICH guidelines. It is clear from this review that future harmonized global guidance needs to focus on clarifying stability testing for OPAT to specifically include temperature requirements, duration of testing requirements, acceptance criteria, degradation products and methods for extrapolation of data. The proposed harmonized document should encompass recommendations from the WHO,^24^ ASEAN,^25^ FDA,^30^ ICH,^26^ EMA,^22^ TGA^23^ and NHS.^10^ The harmonization of the three pharmacopoeial monographs, the BP, EP and the USP, would also be highly useful.^33,35,48^

A harmonized guidance and pharmacopoeia would provide easily accessible and clinically relevant data that are validated and universally accepted to meet regulatory requirements. A precedent has been set by the ICH in deferring to the WHO for clearer and more specific guidance in the past.^24^ However, with the deadline of 2025^29^ already announced by the ICH, it remains to be seen whether a clear consensus between the regulatory authorities globally will emerge. Until further guidance is released, the UK’s NHS YCD promoted by BSAC is the only authority to provide guidance specific to OPAT^8^ and therefore stability studies should draw some level of guidance from these documents.^8,10^ It is essential for clinicians, researchers and pharmaceutical companies to collaborate in addressing the lack of harmonization in antimicrobial stability testing for OPAT. The ICH has already made progress through global harmonization initiatives,^29^ building on evaluations of existing international guidelines. Collaborative research, such as BSAC’s OPAT strategy, has facilitated partnerships among stakeholders to produce evidence aligned with regulatory standards.^9^ To further strengthen this effort, integration with regulatory authorities could create an opportunity for a globally unified framework for stability testing. Leveraging educational programmes like the CRE_RESPOND’s OPAT seminars^52^ and BSAC’s workshops^53^ could also enhance international collaboration and drive forward multinational regulatory efforts.

Future studies should provide more detailed harmonization recommendations for OPAT stability testing. Additionally, a regulatory global framework is needed, particularly in terms of ‘areas of variation’ amongst current guidance, and a pragmatic approach may be required. Further studies should not be limited to the evaluation of stability evidence using stability acceptance criteria from the three pharmacopoeias, but also consider climate differences in geographical regions.

Antimicrobial resistance is recognized as a global priority by the WHO. An important update to the good practice recommendations for OPAT in the UK, published in 2019, drew a clear and significant connection to the role of OPAT in antimicrobial stewardship as well as the importance of obtaining robust and reliable stability data.^51^ A global harmonized stability testing framework for OPAT is vital for this priority.

## Transparency declarations

J.A.R. is a consultant/advisory board member of Qpex (2022), Gilead (2022), Advanz Pharma (2022), Pfizer (2020), Sandoz (2020), Wolters Kluwer (2021), MSD (2021) and Summit Pharma (2021). He has also received funding from Pfizer (2023), MSD (2022), Gilead (2022) and Cipla (2021) to speak at conferences, and received industry grants from Pfizer (2023), QPEX (2021) and the BSAC (2021). F.B.S. has received industry grants from Pfizer and Gilead Australia. M.G. is a consultant/advisory board member for Advanz Pharma (2023), Sandoz (2020), Eumedica (2022) and Pfizer (2023), and has received funding from Menarini and Napp to speak at conferences. R.A.S. served as a consultant and speaker for Advanz, Pfizer, Mundipharm, Napp and Menarini and is a trustee of BSAC as well as co-lead with M.G. of the BSAC initiative. S.N., V.C. and S.L.P., all have none to declare.
